# Supplementary figures and images for: Blood-Based EWAS of Asthma Polygenic Burden in The Netherlands Twin Register
Source: Biomolecules. 2025 Feb 8;15(2):251. doi: 10.3390/biom15020251 (PMC11852504; doi:10.3390/biom15020251)

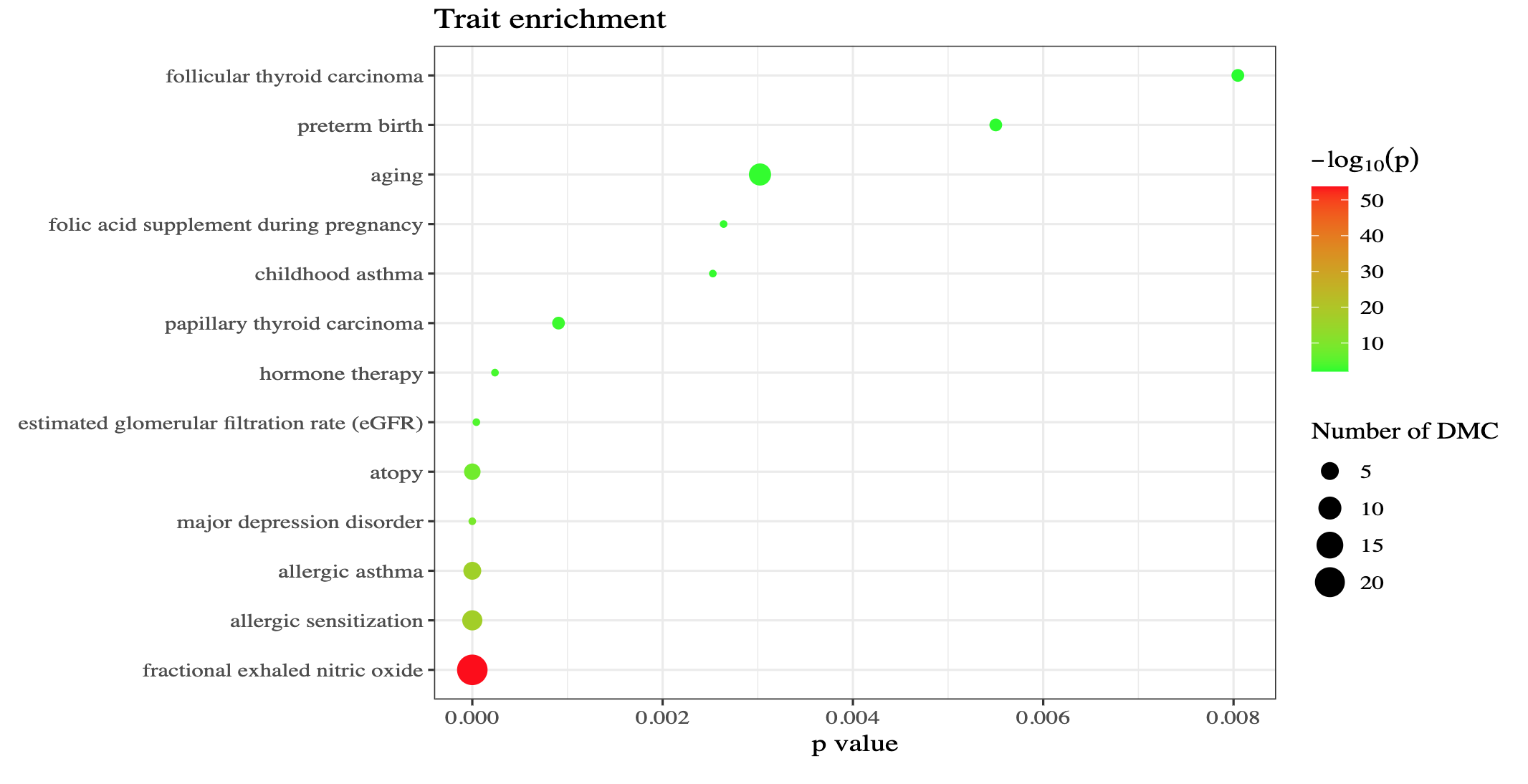

Supplement: Supplementary file 1 [file biomolecules-15-00251-s001.zip › Supplemental Figure S1.png]

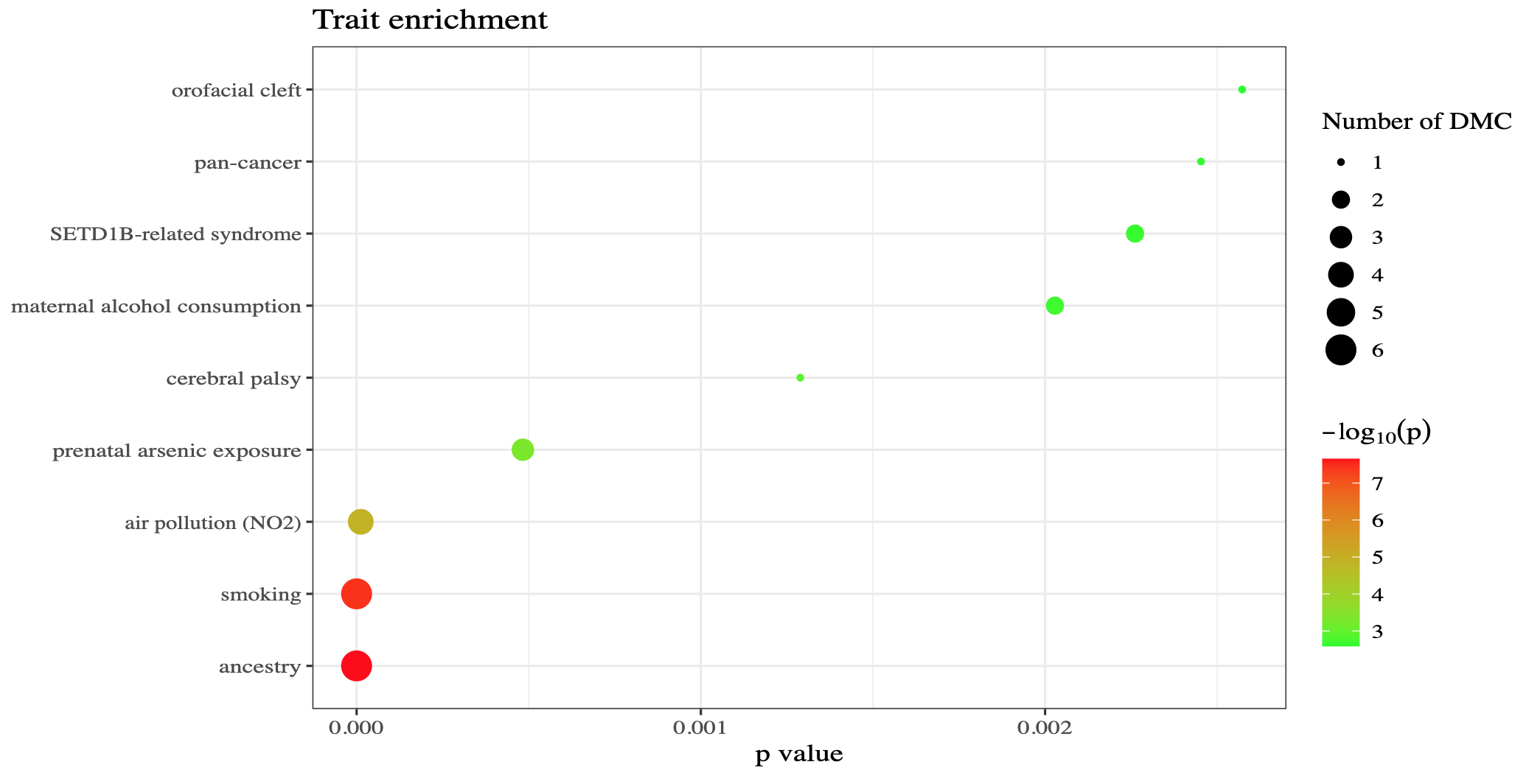

Supplement: Supplementary file 1 [file biomolecules-15-00251-s001.zip › Supplemental Figure S2.png]
